# Exhaled *Mycobacterium tuberculosis* Predicts Incident Infection in Household Contacts

**DOI:** 10.1093/cid/ciac455

**Published:** 2022-11-09

**Authors:** Caroline M Williams, Abdul K Muhammad, Basil Sambou, Adama Bojang, Alhaji Jobe, Georgetta K Daffeh, Olumuyiwa Owolabi, Daniel Pan, Manish Pareek, Michael R Barer, Jayne S Sutherland, Pranabashis Haldar

**Affiliations:** Department of Respiratory Sciences, University of Leicester, Leicester, United Kingdom; Vaccines and Immunology Theme, Medical Research Council Unit The Gambia, London School of Hygiene and Tropical Medicine, Fajara, The Gambia; Vaccines and Immunology Theme, Medical Research Council Unit The Gambia, London School of Hygiene and Tropical Medicine, Fajara, The Gambia; Vaccines and Immunology Theme, Medical Research Council Unit The Gambia, London School of Hygiene and Tropical Medicine, Fajara, The Gambia; Vaccines and Immunology Theme, Medical Research Council Unit The Gambia, London School of Hygiene and Tropical Medicine, Fajara, The Gambia; Vaccines and Immunology Theme, Medical Research Council Unit The Gambia, London School of Hygiene and Tropical Medicine, Fajara, The Gambia; Vaccines and Immunology Theme, Medical Research Council Unit The Gambia, London School of Hygiene and Tropical Medicine, Fajara, The Gambia; Department of Respiratory Sciences, University of Leicester, Leicester, United Kingdom; Department of Respiratory Sciences, University of Leicester, Leicester, United Kingdom; Department of Respiratory Sciences, University of Leicester, Leicester, United Kingdom; Vaccines and Immunology Theme, Medical Research Council Unit The Gambia, London School of Hygiene and Tropical Medicine, Fajara, The Gambia; Department of Respiratory Sciences, University of Leicester, Leicester, United Kingdom

**Keywords:** face mask sampling, tuberculosis, transmission, infectiousness, exhaled breath

## Abstract

**Background:**

Halting transmission of *Mycobacterium tuberculosis* (*Mtb*) by identifying infectious individuals early is key to eradicating tuberculosis (TB). Here we evaluate face mask sampling as a tool for stratifying the infection risk of individuals with pulmonary TB (PTB) to their household contacts.

**Methods:**

Forty-six sputum-positive PTB patients in The Gambia (August 2016–November 2017) consented to mask sampling prior to commencing treatment. Incident *Mtb* infection was defined in 181 of their 217 household contacts as QuantiFERON conversion or an increase in interferon-γ of ≥1 IU/mL, 6 months after index diagnosis. Multilevel mixed-effects logistical regression analysis with cluster adjustment by household was used to identify predictors of incident infection.

**Results:**

*Mtb* was detected in 91% of PTB mask samples with high variation in IS6110 copies (5.3 × 10^2^ to 1.2 × 10^7^). A high mask *Mtb* level (≥20 000 IS6110 copies) was observed in 45% of cases and was independently associated with increased likelihood of incident *Mtb* infection in contacts (adjusted odds ratio, 3.20 [95% confidence interval, 1.26–8.12]; *P* = .01), compared with cases having low-positive/negative mask *Mtb* levels. Mask *Mtb* level was a better predictor of incident *Mtb* infection than sputum bacillary load, chest radiographic characteristics, or sleeping proximity.

**Conclusions:**

Mask sampling offers a sensitive and noninvasive tool to support the stratification of individuals who are most infectious in high-TB-burden settings. Our approach can provide better insight into community transmission in complex environments.

## Teaser

Face mask sampling offers a novel clinical tool that informs infectiousness of pulmonary tuberculosis (TB) more reliably than sputum bacillary load and has the potential to transform public health interventions in high-TB-burden settings.

## INTRODUCTION

Tuberculosis (TB) remains a major global health challenge [1], exacerbated by the coronavirus disease 2019 (COVID-19) pandemic [2]. *Mycobacterium tuberculosis* (*Mtb*) is reliant on airborne transmission, so identifying individuals emitting infectious bacilli is key to interrupting the cycle of infection [3]. Recent studies have demonstrated that tidal breathing may account for >90% of aerosolized *Mtb* [4], potentially generated when small airways reopen during inhalation, rather than by shear forces created by coughing and sneezing [5].

The World Health Organization advocates screening close contacts of pulmonary TB (PTB) cases to identify recently infected individuals as part of the TB elimination strategy [6]. In high-TB-burden settings, systematic screening is resource constrained [7]. Tools that rapidly identify the most infectious individuals could support development of more focused contact screening pathways. However, traditional clinical markers of infectivity including sputum bacillary burden, radiographic disease extent, and cough frequency are known to be unreliable in measures of transmission at the individual level [8–12].

In contrast, direct sampling of aerosolized bacilli from infected individuals using the Cough Aerosol Sampling System (CASS) shows better discrimination between PTB patients with high and low levels of infectiousness than traditional markers [9]. CASS detects colony-forming unit bacilli captured following two 5-minute bouts of coughing. Although effective, the method requires carefully calibrated apparatus, trained personnel, and access to a biosafety level 3 laboratory.

We have developed face mask sampling (FMS) as an alternative method for quantifying bacilli exhaled by PTB patients. The approach is simple, noninvasive, and applicable in any setting where a mask can be worn. We have previously reported individual patterns of *Mtb* emission with FMS over 24 hours and identified dissociation with concomitant sputum bacillary output and cough frequency. As a screening tool for PTB, our studies indicate that FMS can offer advantages over conventional sputum analysis, particularly in nonproductive individuals [13, 14].

In this prospective cohort study, we evaluate the potential of FMS to stratify individual infectiousness of PTB. We hypothesize that emitted bacillary genomic signals detected by FMS are correlated with household contact transmission rates. The work has been conducted in household contacts of PTB patients in The Gambia, where both *Mycobacterium africanum* and *Mtb* cause clinical disease [15].

## METHODS

### Study Population and Design

Sputum acid-fast bacilli (AFB) smear-positive PTB patients were recruited between August 2016 and November 2017, in The Gambia, West Africa. Recruitment was done at the Medical Research Council (MRC) TB clinic (Fajara) and 4 local health centers (Brikama, Fajikunda, Jammeh Foundation for Peace Hospital, and Serrekunda). Patients aged ≥18 years, with at least 3 adult household contacts (HHCs) resident for at least 3 months, were eligible for enrollment to the study. Recruited PTB patients had a baseline chest radiograph (CXR) and provided a 60-minute mask sample prior to commencing treatment.

HHCs were defined as those residing within the same compound as the index case and were stratified by sleeping proximity (see Supplementary Materials for further details). Contacts were eligible for the study if they were aged ≥18 years; had not been treated for TB in the previous 12 months; and had active TB excluded on baseline assessment with medical history, clinical examination, CXR, and where possible, sputum analysis. All recruited HHCs were tested for human immunodeficiency virus (HIV) at baseline and had blood taken for QuantiFERON TB Gold-in-Tube testing (QFT, Qiagen, Germany) at enrollment and 6 months. No treatment was given to patients identified with latent TB infection during the course of the study.

### Sputum Processing

Processing of the initial screening sputum sample for AFB analysis was performed at local clinics. Confirmatory AFB smear microscopy, Xpert MTB/RIF assay (Cepheid), and liquid culture (BACTEC Mycobacteria Growth Indicator Tube [MGIT] 960, Becton Dickinson) analyses were processed at the MRC Unit microbiology laboratory. Mycobacterial species was determined by spoligotyping [16], and radiologic extent of disease was graded on a 4-category ordinal scale independently by 2 members of the research team (Supplementary Table 1).

### FMS and Processing

**Figure 1.**
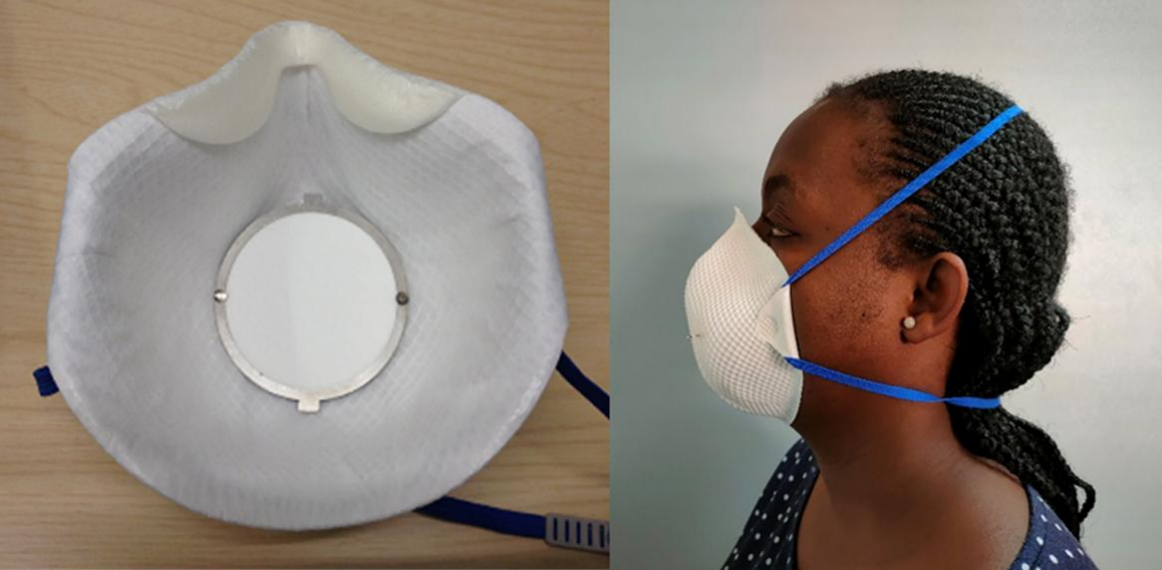
Face mask sampling tool. FFP1 mask containing gelatin filter removed for analysis.

Index cases wore a modified face mask (Moldex 2380 FFP1 NR D) containing a gelatin sampling matrix (diameter 60 mm, pore size 0.3 µm, Sartorius, Germany; catalog number 12602-80-ALK) for 1 hour under direct observation and *Mtb* DNA was extracted and quantified, as previously described [13] (Figure 1). In brief, exposed gelatin from the face mask was dissolved in sodium hydroxide (1.5 mL of 2% w/v), neutralized with 190 μL 4 mol/L hydrochloric acid, and centrifuged at 13 400*g* for 10 minutes; then the pellet was resuspended in Tris–ethylenediaminetetraacetic acid buffer and stored at −80°C. Cells were subsequently disrupted by bead-beating and DNA extraction as previously described [13]. Bacillary burden was assayed by IS6110-directed polymerase chain reaction [17].

### QFT Analysis

At baseline and 6 months, whole blood was collected from HHCs and tested for immunoreactivity to *Mtb* antigens using the QFT assay (1 mL per tube for TB-Antigen, Nil, and Phytohaemagglutinin (PHA)) in accordance with the manufacturer’s instructions. Data were analyzed using the manufacturer’s recommended positive cutoff of ≥0.35 IU/mL and ≥25% of the Nil response after subtracting the Nil from each antigen-specific response.

### Statistical Analysis

Our primary exposure variable was mask bacterial load from the index case and this was correlated with infection in their HHCs. Following the approach described in the CASS study of Jones-Lopez et al [9] and based on the trend in risk of QFT conversion associated with finely divided categories of IS6110 copy numbers, we divided mask output into 2 groups (≥20 000 copies; <20 000 copies or negative); these were used for further analyses.

Our primary outcome measure was incident *Mtb* infection within HHCs, defined by QFT conversion from negative to positive. Our secondary outcome measure used a quantitative increase of ≥1 IU/mL in QFT from baseline to 6 months, regardless of baseline IFN-γ result. This secondary measure was included to overcome data loss from missed QFT conversion events arising from delayed index diagnosis. A threshold increment of ≥1 IU/mL was chosen as studies indicate this threshold associates with a significant infection event [18, 19] and TB disease progression [20, 21].

We calculated a negative predictive value (NPV) of FMS to predict incident infection (QFT conversion and IFN-γ increase of ≥1 IU/mL) with an average transmission rate to household contacts of 29% [22].

We calculated descriptive statistics of clinical and demographic characteristics of contacts of index cases and compared differences between subgroups with those exposed to high mask output and low-positive/negative mask output. Factors associated with incident *Mtb* infection in contacts were determined using multilevel mixed-effects logistic regression, adjusting for clustering within households. Variables included to the model were selected based on achieving a statistical threshold of *P* < .1 in univariable analysis (Supplementary Table 4). Statistical analyses were performed using Stata version 13.1 software. Results are presented as unadjusted and adjusted odds ratios (ORs and AORs, respectively) with 95% confidence intervals (CIs).

### Ethics Statement

Written informed consent was obtained from all participants prior to sample collection. Ethical approval was provided by The Gambia Government/MRC joint ethics committee (reference number SCC 1486v2).

## RESULTS

**Figure 2.**
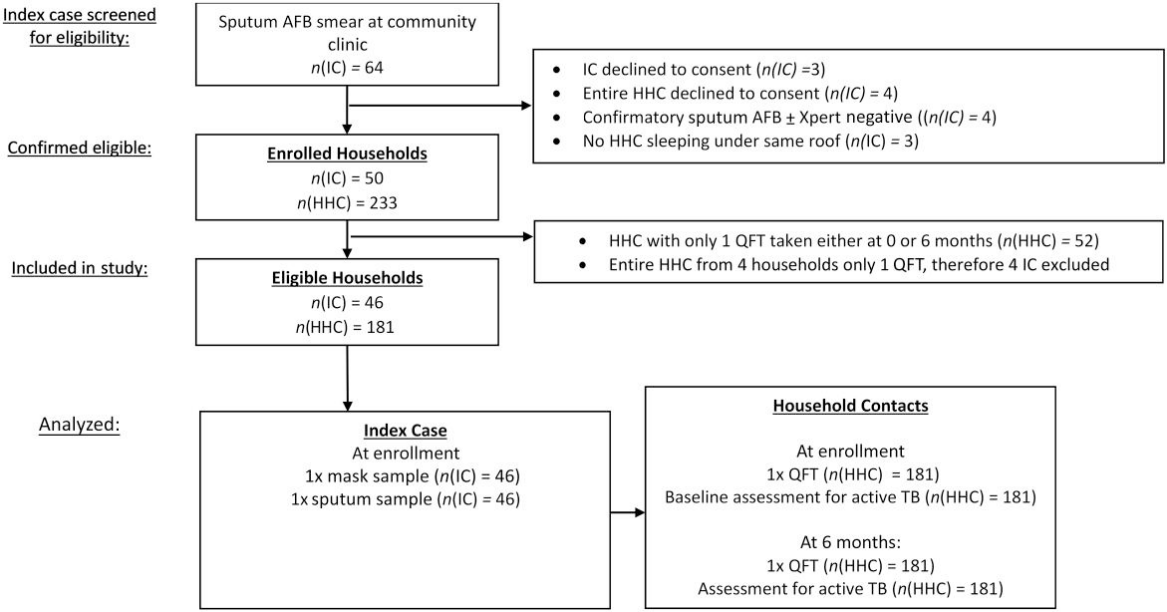
Study profile. Abbreviations: AFB, acid-fast bacilli; HHC, household contact; IC, index case; QFT, QuantiFERON Gold-in-Tube assay; TB, tuberculosis.

Between February 2017 and May 2018, we screened 64 sputum AFB smear-positive PTB patients and enrolled 50 participants (Figure 2). Four index cases and their HHCs were later withdrawn because all HHCs were lost to follow-up or had missing results. In total, 181 of 217 (85%) HHCs were included from households of 46 index cases (Figure 2). There was no difference in baseline characteristics between TB cases and HHCs that were included and excluded from the study (Supplementary Tables 2 and 3).

### TB Cases and Mask Output

All 46 index PTB cases had microbiologically confirmed disease, with 14 (30%) having *M. africanum* infection (Table 1). Median age of the cohort was 26 years, and 27 participants (59%) were male. HIV coinfection was identified in 2 participants (4%). All participants reported cough and other TB-associated clinical symptoms for >3 weeks prior to enrollment and all had moderate or advanced disease on CXR.

**Table 1.** Tuberculosis Case Demographics and Clinical, Radiological, and Microbiological Characteristics, Stratified by Mask-Captured IS6110

Characteristic	Mask Negative/Low Positive^a^	Mask High Positive^b^	Total Cohort	*P* Value
No.	27	19	46	.43
Age, years, median (IQR)	23 (20–33)	30 (21–48)	26 (20–40)	.19
Male sex	17 (63)	10 (53)	27 (59)	.48
BMI, kg/m^2^, median (IQR)	18 (17–19)	18 (17–21)	18 (17–19)	.72
TB symptoms >3 weeks prior to enrollment	27 (100)	19 (100)	46 (100)	1.0
HIV positive	0 (0)	2 (10)	2 (4)	.08
CXR findings				
ȃExtent of disease				
ȃȃNormal	0	0	0	.31
ȃȃMinimal	0	0	0	
ȃȃModerate	4 (15)	0	4 (9)	
ȃȃAdvanced	23 (85)	19 (100)	42 (91)	
ȃPresence of cavities				
ȃȃYes	8 (30)	8 (42)	16 (35)	.38
ȃȃSize of cavity if present^c^, cm, median (IQR)	4 (4–5)	4 (3–5)	4 (2–6)	.60
Sputum characteristics				
ȃAFB smear				
ȃȃNegative	0	0	0	.60
ȃȃ1+	8 (30)	7 (37)	15 (33)	
ȃȃ2+	10 (37)	5 (26)	15 (33)	
ȃȃ3+	9 (33)	7 (37)	16 (34)	
ȃXpert MTB/RIF				
ȃȃNegative	0	0	0	.15
ȃȃLow	6 (22)	3 (16)	9 (20)	
ȃȃMedium	12 (45)	9 (47)	21 (46)	
ȃȃHigh	9 (33)	7 (37)	16 (35)	
ȃRifampicin resistance present	1 (4)	0	1 (2)	.40
ȃMGIT 960 culture^d^ (DTP), median (IQR)	11 (8–14)	7 (6–11)	12 (4–40)	.85
Mycobacterial species				
ȃ*M. tuberculosis*	21 (78)	12 (63)	33 (72)	.89
ȃ*M. africanum*	6 (22)	7 (37)	13 (28)	

Data are presented as No. (%) unless otherwise indicated.

Abbreviations: AFB, acid-fast bacilli; BMI, body mass index; CXR, chest radiograph; DTP, days to positivity; HIV, human immunodeficiency virus; IQR, interquartile range; MGIT, Mycobacteria Growth Indicator Tube; TB, tuberculosis.

Mask negative/low positive: IS6110 copies <20 000.

Mask high positive: IS6110 copies ≥20 000.

n = 18 for cavities present on CXR.

Missing data for MGIT 960 culture: mask negative/low positive (n = 3); mask high positive (n = 3); whole cohort (n = 6).

Exhaled *Mtb* was detected by FMS in 42 (91%) index cases with IS6110 copy numbers varying from 5.3 × 10^2^ to 1.2 × 10^7^ (median, 1.8 × 10^4^) among mask-positive individuals. Nineteen participants (45%) had a high mask output (>20 000 copies).

Table 1 shows that the high mask output group did not differ significantly from the negative/low mask output group on sputum analyses by smear AFB (*P* = .60), Xpert MTB/RIF (*P* = .15) grades, time to positive culture with MGIT 960 (*P* = .85), CXR severity (*P* = .31), or proportion with cavitation (*P* = .38). Index cases with *M. africanum* infection were similarly distributed between the high-positive and low-positive/negative mask output groups (37% vs 22%; *P* = .89).

### Household Contacts and Prevalent TB Infection

The number of HHCs exposed to TB patients with negative, low-positive, and high-positive mask samples were 10, 98, and 73 respectively. No HHCs were found to have active TB at baseline. The number of HHCs exposed to mask-negative patients was low (n = 4), so this was combined with those associated with low-positive mask samples for comparative analyses with contacts of high-positive mask output index cases. The groups were statistically matched according to sleeping proximity to their index case (*P* = 0.15), evidence of BCG vaccination (*P* = 0.114), and baseline prevalence of QFT-defined latent *Mtb* infection (53% for index mask-negative/low-positive vs 52% for index high-positive mask output contacts; *P* = 0.933) (Table 2).

**Table 2.** Household Contact Characteristics, Stratified by Index Case Exhaled *Mycobacterium tuberculosis* Measured by Mask Sampling Using IS6110

Characteristic	Mask Negative/Low Positive^a^	Mask High Positive^b^	Total Cohort	*P* Value
No.	108	73	181	.46
Age, years, median (IQR)	25 (20–36)	26 (20–40)	25 (20–40)	.476
Male sex	39 (36)	28 (38)	70 (39)	.968
HIV positive	1 (1)	3 (4)	4 (2)	.692
BMI, kg/m^2^, median (IQR)	22 (19–27)	20 (19–25)	21 (19–25)	.300
BCG scar present	57 (53)	3 (45)	90 (50)	.114
Sleeping proximity to index case				
ȃSame room	18 (16)	14 (19)	32 (18)	.152
ȃDifferent room, same house	59 (55)	50 (68)	109 (60)	
ȃSame household, different hut/house	31 (29)	9 (12)	40 (22)	
QFT positivity				
ȃBaseline	56 (53)	38 (52)	94 (52)	.933
ȃ6 months	56 (53)	44 (60)	100 (55)	.295
ȃIGRA conversion (neg to pos)	15 (29)	19 (54)	34 (39)	.043
Quantitative QFT result ≥1 IU/mL positive	16 (15)	25 (34)	41 (23)	.005

Data are presented as No. (%) unless otherwise indicated.

Abbreviations: BMI, body mass index; HIV, human immunodeficiency virus; IGRA, interferon-γ release assay; IQR, interquartile range; QFT, QuantiFERON TB Gold-in-Tube assay.

Mask negative/low positive: IS6110 copies <20 000.

Mask high positive: IS6110 copies ≥ 20 000.

### Incident *Mtb* Infection

#### Primary Outcome Measure (QFT Conversion)

**Figure 3.**
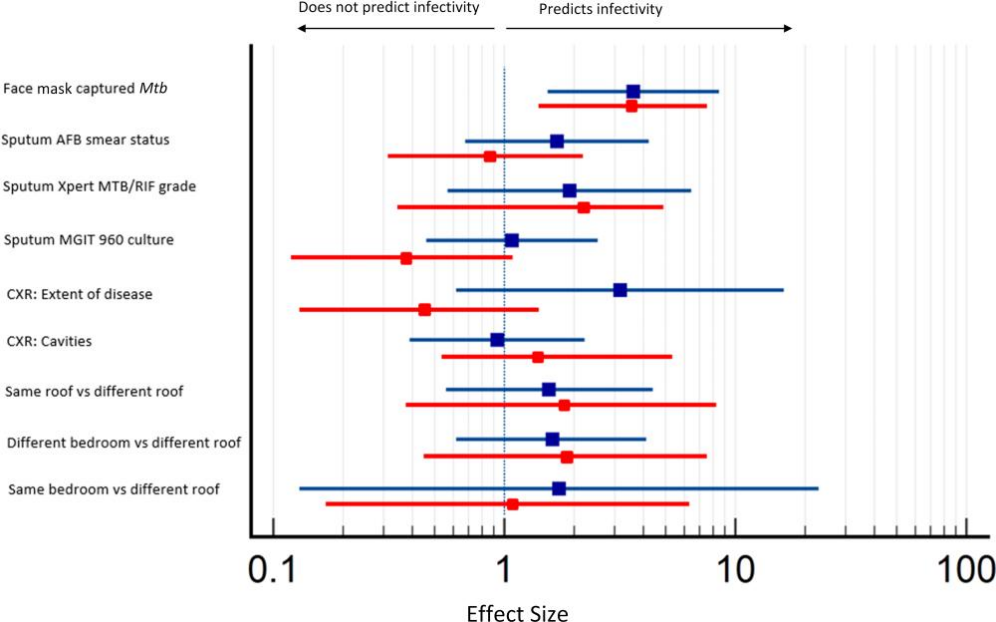
Adjusted odds ratios for predictors of transmission associated with 2 infection outcome measures: QuantiFERON (QFT) conversion (red) or QFT change +1 IU/mL or greater (blue) in exposed household contacts. Abbreviations: AFB, acid-fast bacilli; CXR, chest radiograph; MGIT, Mycobacteria Growth Indicator Tube; *Mtb*, *Mycobacterium tuberculosis*.

After excluding HHCs who were QFT positive at baseline, there were 41 (47%) contacts of low-positive or mask-negative output and 46 (53%) contacts of high-positive mask output index cases available for assessment of QFT conversion at 6 months. Nineteen QFT conversions (26%) occurred in contacts of high-positive mask output index cases, compared with 14 (14%) in household contacts of low-positive and 1 in contacts of mask-negative index cases. In our logistic regression model, contacts of high-positive mask output cases were at significantly greater risk of incident *Mtb* infection, compared with contacts of low-positive and negative-mask output cases (AOR, 3.20 [95% CI, 1.26–8.12]; *P* = 0.01) (Figure 3 and Table 3). The calculated NPV of FMS for incident infection was 75.5% (95% CI, 71.8%–85.5%).

**Table 3.** Adjusted Odds Ratios for Predictors of Transmission Associated With 2 Infection Outcome Measures: QuantiFERON (QFT) Conversion or QFT Change +1 or Greater in Exposed Household Contacts

HHC Infection Predictor	QFT Conversion (Negative to Positive)			QFT Change ≥ +1 IU/mL		
	AOR	(95% CI)	*P* Value	AOR	(95% CI)	*P* Value
Face mask captured *Mtb*^a^	3.20	(1.26–8.12)	.01	3.26	(1.54–8.53)	.003
Sputum *Mtb* burden						
ȃAFB smear status^b^	0.82	(.30–2.20)	.69	1.69	(.68–4.22)	.26
ȃXpert MTB/RIF grade^c^	1.11	(.33–3.73)	.86	1.92	(.57–6.45)	.29
ȃMGIT 960 culture^d^	0.38	(.13–1.09)	.07	1.08	(.46–2.54)	.85
Chest radiograph changes						
ȃExtent of disease	0.49	(.13–1.79)	.28	3.17	(.62–16.22)	.17
ȃPresence of cavities	1.45	(.52–4.01)	.48	0.93	(.39–2.23)	.88
Sleeping proximity of HHCs						
ȃSame roof (bedroom or house) vs different roof	1.75	(.38–8.03)	.47	1.56	(.56–4.39)	.39
ȃDifferent bedroom same roof vs different roof	1.84	(.43–7.81)	.41	1.61	(.62–4.13)	.32
ȃSame bedroom vs different roof	1.06	(.17–6.82)	.95	1.72	(.13–22.91)	.68

Abbreviations: AFB, acid-fast bacilli; AOR, adjusted odds ratio; CI, confidence interval; HHC, household contact; MGIT, Mycobacteria Growth Indicator Tube; *Mtb*, *Mycobacterium tuberculosis*; QFT, QuantiFERON Gold-in-Tube assay.

IS6110: ≥20 000 copies vs <20 000 copies or negative.

AFB: 1+ vs >1+.

Low vs higher than low.

Days to positivity: 0–10 vs >10 days and negative.

Measures of sputum *Mtb* burden (AFB grade, Xpert grade) radiological extent of disease and sleeping proximity showed no significant association with QFT conversion in our models (Figure 3 and Table 3). However, a possible association between days to positivity (DTP) in MGIT culture and QFT conversion that approached statistical significance was observed (*P* = .07) (Figure 3 and Table 3).

#### Secondary Outcome Measure: IFN-γ Increase of ≥1 IU/mL at 6 Months

Applying this criterion to the full cohort of 181 HHCs, 25 (34%) new or recently acquired *Mtb* infections occurred in contacts of high IS6110 mask output TB cases, compared with 16 (15%) *Mtb* infections in contacts of low-positive/negative IS6110 index cases. Mask-negative individuals accounted for 1 of the 16 infections within the mask-negative/low-positive group. Using this threshold increase in QFT, contacts of high-output mask cases were again found to have a significantly increased risk of incident *Mtb* infection of comparable magnitude (AOR, 3.62 [95% CI, 1.54–8.53]; *P* = 0.003) (Table 3). The calculated NPV for FMS for incident infection was 80.5% (95% CI, 73.4%–86.0%). Sputum *Mtb* burden, radiological disease, and sleeping proximity were not statistically associated with incident *Mtb* infection (Figure 3 and Table 3). Finally, we observed no difference in the proportion of contacts with newly acquired infection defined by either criterion, according to the bacterial strain of the index case (QFT conversion: *P* = 0 .28; IFN-γ ≥1 IU/mL: *P* = 0.22).

## DISCUSSION

Our data support utility of FMS as a simple and noninvasive clinical tool for stratifying risk of *Mtb* transmission to HHCs in a high-burden setting more effectively than indices of infectivity widely applied in clinical practice. The conduct of this study within the well-established and extensively characterized TB Case-Control (TBCC) platform at MRC Fajara [23] enhances confidence in our findings.

FMS detected exhaled *Mtb* in a large proportion (91%) of the index cases, and HHCs of high-positive FMS output cases had a >3-fold increased likelihood of incident *Mtb* infection based on QFT conversion after 6 months. This stratification of risk was not demonstrated with either sputum bacillary burden or radiological extent of disease, which are indices currently used by clinicians for this purpose [24]. Consistent with previous reports, we also found no difference in disease severity, FMS mycobacterial output, or transmission in contacts of indexes infected with *M. africanum* (28% of our cohort) [25].

Transmission studies are limited by absence of a reliable objective biomarker for this outcome. We used QFT conversion as our primary endpoint. This approach is supported by studies that have demonstrated increased risk of progression to TB in those with QFT conversion, an outcome associated with recent infection [20, 26, 27]. While recognition of incident infection resulting from household transmission is inferred from QFT conversion here, we accept that this could also reflect infection acquired prior to index diagnosis or from another source. QFT conversion here was assessable in 87 (48%) HHCs, while in the CASS transmission study [9] only 27% of HHCs could be included for this analysis, emphasizing the need for better and more inclusive measures of transmission. We included a significant quantitative increase in QFT [18–21] as a secondary outcome measure of transmission, to allow inclusion of our complete HHC cohort in analyses. The consistency of results observed between these 2 measures of transmission support consideration of quantitative changes in interferon (IFN)–γ release assay (IGRA) response for future studies.

As a tool measuring exhaled bacilli, FMS is comparable to CASS, with notable similarities and differences in outcomes. Both FMS and CASS output associate significantly with incident *Mtb* infection in HHCs in a manner that is not observed for traditional markers of infectivity, including sputum bacillary burden and radiological extent of disease [8–10, 28–30]. Furthermore, our previous FMS studies identified inconsistencies in the relationship between mask and sputum bacillary burden, which is also reported by aerosol studies [9, 13, 29, 31–33]. These observations support the view that exhaled and aerosolized *Mtb* constitute a distinct *Mtb* pool, more strongly associated with transmission than other measures of bacillary burden [30]. However, we note some important differences between FMS and CASS. First, there is a significant difference in the proportion of *Mtb*-positive individuals identified, which may influence the stratification of transmission risk. *Mtb* was detected in 91% of PTB patients sampled with FMS, compared with 45% using CASS [7]. In that study, a >9-fold increased odds of IGRA conversion in HHCs of patients with a high aerosol output was reported, compared with the 3-fold difference in odds identified using FMS. However, 36% of the contacts of index cases with a negative CASS and 47% of the contacts of cases with a low *Mtb* CASS output had evidence of recent transmission. In contrast, we found that only 14% of contacts of negative or low-positive FMS *Mtb* output PTB cases had QFT conversion. This suggests that while CASS-measured *Mtb* output is more specific, it is considerably less sensitive than FMS for informing transmissibility of infectious PTB. Furthermore, the high NPV of FMS (79.5%–80.5%) suggests that a negative result with FMS could usefully inform low-risk contacts in resource-limited settings. The differences between FMS and CASS may reflect differences in captured material. CASS only identifies culturable bacilli, which is likely to be a minor subset of the *Mtb* DNA–positive material detected with FMS, including free DNA and nonreplicating cells.

This study has some limitations. It was not powered to assess the association between existing clinical measures of infectivity such as sputum bacillary load, sleeping proximity, and radiographic changes and transmission, as evidenced by the relatively large CIs observed in analyses of these factors and a near-significant association of DTP in MGIT culture with QFT conversion. Larger studies are needed to reliably investigate these factors, including systematic cough recording, which has not been addressed here. Nevertheless, the consistency of our findings with previous aerosol transmission studies supports a stronger association of transmission with *Mtb* populations captured in exhaled air. A larger study using FMS would allow more nuanced evaluation of the association between mask output and transmitted infection. In particular, it would retain power after inherent data loss to examine transmission cofactors, enable more granular statistical analyses using multiple mask output thresholds, and allow specific investigation of FMS-negative, sputum-positive index cases, which were too small in number in our cohort to analyze independently. The low HIV/TB coinfection rate in The Gambia limits generalizability of our findings to other high-TB-burden areas, due to potential influence of HIV coinfection on both index case infectivity and susceptibility of contacts. However, the proportion of FMS-positive patients in this study is comparable with our previously published work, which was in a predominately HIV-positive cohort [13]. Further work in settings of high HIV, high TB burden and low TB burden are needed to fully assess the generalizability of mask sampling as a clinical infectivity tool. Our method for measuring transmission has weaknesses but remains the best measure available. Including tuberculin skin test with QFT could have identified additional cases of incident infection and provided comparative data for analysis. We acknowledge that the lack of a population control and 6-month interval between QFT tests used in this study [9, 12, 34] increases uncertainty of *Mtb* exposure attribution to the identified source case. We did not perform FMS in the contact cohort and are therefore unable to determine whether any cases of transmissible subclinical infection could have been identified. Finally, we were unable to include children in our study due to a worldwide shortage of tuberculin and logistical limitations of TBCC at the time. Future studies should aim to recruit children and consider measuring infections with >1 assay.

In summary, FMS is a noninvasive, inexpensive, and easily deployable tool that demonstrates capability to stratify transmission risk from individuals with PTB. This builds on our previous work supporting a role for mask sampling as a diagnostic tool in those with both sputum smear-positive and smear-negative disease [13]. The influence of the COVID-19 pandemic on mask-wearing behavior, especially within clinical settings, offers a natural opportunity for FMS to be integrated into clinical practice. Our studies support the potential of FMS as a clinical tool to enhance TB control programs necessary for eradication of TB [35] and provides an epidemiological tool to better characterize *Mtb* transmission within community settings.

## Supplementary Material

ciac455_Supplementary_Data

## Supplementary Data

Supplementary materials are available at *Clinical Infectious Diseases* online. Consisting of data provided by the authors to benefit the reader, the posted materials are not copyedited and are the sole responsibility of the authors, so questions or comments should be addressed to the corresponding author.

## Notes

***Financial support.*** This work was supported by the Wellcome Trust (grant number 107302/Z/15/Z to C. M. W.).
